# Visualization of Internal Working Models Through Transactional Analysis (TA) Developmental Collage Therapy: A Case Report

**DOI:** 10.7759/cureus.102599

**Published:** 2026-01-29

**Authors:** Hidemi Nakano

**Affiliations:** 1 Clinical Psychology, Tokyo Clinical Psychology Counseling Academy, Tokyo, JPN

**Keywords:** art-therapy, attachment, case report, collage therapy, ego states, internal working model, life script, non-verbal psychotherapy, pre-verbal trauma, transactional analysis

## Abstract

Bowlby's Internal Working Model (IWM) is central to understanding adult attachment-related difficulties. However, what IWM concretely consists of remains unspecified, and no method exists for directly visualizing IWM. Because IWM is formed during the pre-verbal period, it is difficult to access through language-based psychotherapy alone. A woman in her 20s with a history of childhood stuttering and subsequent interpersonal difficulties participated in Transactional Analysis (TA) Developmental Collage Therapy. She created six collage works representing "myself and my surrounding environment" across developmental stages from birth to a future after therapeutic change. Pre- and post-intervention assessments included the Tokyo University Egogram Third Edition (TEG3), which measures five ego states based on TA theory, and the Experiences in Close Relationships inventory-Generalized Other version (ECR-GO), which assesses attachment anxiety and avoidance. The collage works visually depicted the relationship between the "Child" ego state (Free Child or Adapted Child) and the surrounding environment ("Parent") at each developmental stage. Collages 1-2 expressed stable attachment with Free Child; Collages 3-4 depicted transformation to insecure attachment with Adapted Child following negative experiences; Collages 5-6 showed re-emergence of Free Child and movement toward secure attachment. TEG3 showed decreased Critical Parent (CP: 49→35) and Adapted Child (AC: 50→45), with increased Free Child (FC: 57→59). ECR-GO showed decreased Attachment Anxiety (54→48) and Attachment Avoidance (51→45). This case suggests that TA Developmental Collage Therapy can visualize IWM and facilitate its transformation. The findings indicate that ego states may constitute structural components of IWM, and that combining non-verbal collage techniques with verbal approaches may provide an effective therapeutic framework that complements language-based psychotherapy.

## Introduction

Bowlby [1] proposed attachment theory, describing the emotional bond formed between infants and their caregivers as "attachment." Inadequate attachment formation during infancy has serious effects on child development. The Diagnostic and Statistical Manual of Mental Disorders, Fifth Edition (DSM-5) includes diagnostic criteria for childhood attachment disorders: Reactive Attachment Disorder (RAD) and Disinhibited Social Engagement Disorder (DSED) [2].

In recent years, clinical attention has increasingly focused on attachment-related problems in adults. Adults presenting with a wide range of difficulties, including interpersonal problems, emotional dysregulation, negative self-concept, and somatic symptoms, often have underlying issues related to inadequate attachment formation in early childhood [3-5]. The introduction of complex post-traumatic stress disorder (PTSD) in the International Classification of Diseases, 11th Revision (ICD-11) acknowledged that early relational trauma can have lasting effects into adulthood [6]. However, there remains no established diagnostic category that directly addresses attachment problems in adults, and standardized treatments have not been developed.

Central to understanding these adult attachment-related difficulties is Bowlby's concept of the Internal Working Model (IWM). Bowlby [3] defined IWM as a structure comprising two complementary models - a model of the attachment figure and a model of the self - that generates forecasts about the availability and responsiveness of the attachment figure and one's own acceptability. Furthermore, Bowlby stated that adult personality is "a product of an individual's interactions with key figures during all his years of immaturity, especially of his interactions with attachment figures" [3]. IWM is thought to be formed through interactions with caregivers during infancy and to continue regulating interpersonal relationships in adulthood [3,5].

Despite the clinical importance of IWM, several fundamental questions remain unresolved. Bowlby provided only a conceptual definition of IWM; what IWM concretely consists of has not been specified. It remains unclear how IWM is formed, why it resists change, and through what mechanisms it can be modified. Furthermore, because IWM is formed during the pre-verbal period and operates largely through implicit memory systems in the right hemisphere [4], it is inaccessible through language-based psychotherapy and cannot be verbalized by the individual. No established method exists for directly observing or measuring IWM.

The following section explains the key concepts used in this study. Transactional analysis (TA) is a theory of personality and a systematic psychotherapy for personal growth and change [7]. TA provides a theory of personality (the ego-state model), a theory of communication, a theory of child development (the life-script concept), and a theory of psychopathology [7].

Berne [8] defined ego states as "a set of feelings, attitudes, and behavior patterns." There are three main categories. The Parent (P) ego state is a set of feelings, attitudes, and behavior patterns that resemble those of a parental figure [8]. Functionally, it is divided into Critical Parent (CP), which enforces rules and values and provides criticism and guidance, and Nurturing Parent (NP), which provides caring, protection, and permission [7]. The Adult (A) ego state is an autonomous set of feelings, attitudes, and behavior patterns that are adapted to the current reality [8]. The Child (C) ego state is a set of feelings, attitudes, and behavior patterns that are relics of the individual's own childhood [8]. Functionally, it is divided into Free Child (FC), which represents spontaneous, creative, and natural expression in a stress-free state, and Adapted Child (AC), which represents a state of adapting to the external environment [7]. When AC functions appropriately and can flexibly alternate with FC according to situational demands, this is a healthy adaptation. However, when AC becomes fixed and over-adapted, unable to alternate with FC, psychological difficulties, including anxiety, depression, and interpersonal problems, may arise.

Berne [8] defined the life script as "a life plan based on a decision made in childhood, reinforced by the parents, justified by subsequent events, and culminating in a chosen alternative." Injunctions are defined structurally as "parental prohibitions" transmitted from the parent's child ego state, and functionally as "unreasonable stoppers" enforced by fear [8]. These prohibitions are often internalized as the core of the life script.

Shinoki [9] developed TA Developmental Collage Therapy and demonstrated that when participants created collage works based on instructions derived from TA theory of ego states and life scripts, their life scripts could be visualized and thereby modified.

TA Developmental Collage Therapy shares a common therapeutic mechanism with the empty chair technique used in Redecision Therapy [10]. Both techniques activate specific ego states through instructions specifying a particular context (developmental stage or situation), with the aim of externalizing them so that clients can gain awareness of the specific functioning of the ego states that constitute their personality.

However, there are important differences between the two approaches. First, Redecision Therapy is grounded in verbal redecision. Since making a decision inherently requires linguistic and cognitive maturity, this approach has a structural limitation in addressing the pre-verbal period (0-3 years), where experiences are encoded somatically rather than linguistically. In contrast, TA Developmental Collage Therapy, as a non-verbal approach, can access ego states from the pre-verbal period when attachment is formed. Second, because collage works remain as physical artifacts, clients can revisit their works repeatedly, providing an ongoing opportunity for mentalization. Third, the structure of sequentially tracing developmental stages enables visual tracking of the formation and transformation process of IWM and life scripts.

TA has traditionally focused on analyzing interpersonal transactions - exchanges between individuals [8]. In contrast, attachment theory focuses on how attachment behaviors between parent and child become represented, through developmental processes, as the relationship between the self and the attachment figure within an individual's mind [3]. That is, in contrast to external attachment behaviors between parent and child, the internal relationship between the "P" ego state (the represented model of caregivers) and the "C" ego state (the model of self) can be understood as an internal "attachment representation."

The theoretical integration of attachment theory and TA has been established by several researchers. Erskine [5] proposed that Bowlby's attachment theory provides a theoretical integration with script theory, noting that IWMs are "the antecedents of an unconscious life script." This integration is supported by the temporal overlap in their formation: both IWM and script protocol develop primarily during the pre-verbal period (0-3 years). Furthermore, both theories emphasize the forecasting function of early relational schemas. Bowlby [3] described IWM as generating predictions about attachment figure availability, while Berne [8] described the script as a "life plan" that predicts interpersonal outcomes. This convergence suggests that ego states, as the structural components of TA personality theory, may constitute the building blocks of IWM. Boholst et al. [11] empirically examined the relationship between attachment styles and TA life positions, finding significant correlations that suggest conceptual parallels between the two constructs. The present study extends this theoretical integration by applying it to the internal P-C relationship, defining it as the structural substrate of attachment.

Collage therapy has been defined as a method of "giving form to unconscious images of the inner mind" using ready-made pictures and photographs [12]. TA Developmental Collage Therapy instructs clients to express "yourself and the environment surrounding you" at each developmental stage through collage [9]. This instruction directly corresponds to Bowlby's definition of IWM as comprising "a model of the attachment figure" (environment/other) and "a model of the self" [3]. The collages produced can therefore be understood as visual externalizations of the IWM structure at each developmental stage.

If life scripts can be visualized and modified through collage creation, and if the internal P-C relationship constitutes the structural basis of IWM, then IWM itself may be amenable to visualization and modification through this method. Because IWM exists in the unconscious and is difficult to visualize, its definition has remained unclear, arguably impeding theoretical and clinical advances. The purpose of this case report is to reanalyze collage works created through TA Developmental Collage Therapy within the framework of attachment theory, through a single case, in order to clarify the components of IWM and to verify that IWM can be visualized and modified, using changes in attachment-related measures (the Experiences in Close Relationships inventory-Generalized Other version (ECR-GO)) and ego state measures (the Tokyo University Egogram Third Edition (TEG3)). If this study provides a clue to clarifying the components of IWM and visualizing its process of change, it may contribute to further development of attachment theory and its clinical applications.

Research aims and methodological note

This case report aims to demonstrate proof of concept: that IWM components can be visualized and structurally identified through developmental stage collages. Because attachment theory has not defined IWM's structural components, this research tests whether TA concepts (ego states) can be applied to conceptualize IWM structure, using Erskine's [5] theoretical integration as the foundation.

## Case presentation

This study was conducted as part of a doctoral research project at Kyushu Sangyo University involving 31 healthy adult participants (seven males, 24 females; age range: 25-72 years) [9]. The participants were recruited via convenience sampling from attendees of a three-day (total 21 hours) TA seminar series. Following an initial briefing, an orientation session was held via Zoom online meetings (Zoom Video Communications, San Jose, CA, USA) for those interested. The inclusion criteria were adults who provided written informed consent and were not currently receiving psychiatric treatment. The exclusion criteria included inability to complete the collage process; accordingly, two individuals who found the process difficult were excluded, leaving 31 participants who completed the study. The study protocol was approved as part of the doctoral research process at Kyushu Sangyo University. In accordance with the university's ethical guidelines, all participants were provided with a comprehensive explanation of the study’s purpose, the voluntary nature of participation, the right to withdraw, and the protection of personal information. Written informed consent was obtained from all participants prior to the intervention.

The intervention, TA Developmental Collage Therapy, was implemented between September and December 2020. The following describes the intervention method, followed by the presentation of a single case (Case F) selected from the study. Case F was purposively selected for this presentation because her collage works and accompanying narratives provided the most clinically rich and theoretically transparent illustration of the structural transformation of the IWM. Her case serves as a representative example that clearly demonstrates the mechanism of change within the TA Developmental Collage Therapy framework.

The intervention followed a structured process consisting of five distinct phases, designed to facilitate non-verbal access to the pre-verbal IWM and promote its structural reorganization (Table 1).

**Table 1:** Structure and therapeutic goals of TA developmental collage therapy TA: Transactional Analysis; TEG3: Tokyo University Egogram Third Edition; ECR-GO: Experiences in Close Relationships inventory-Generalized Other version

Phase	Title	Duration/Format	Primary Therapeutic Goal and Mechanism
1	Orientation	120 min (Group/Online)	Establishing the "Adult" (A) contract. Instructions for visualizing "Self and Environment" to target the IWM structure.
2	Individual Counseling	45 min (Individual)	Pre-analysis of developmental history and life scripts. Establishing a therapeutic alliance (External Secure Base).
3	Pre-intervention Assessment	Home-based	Quantitative baseline of Ego States (TEG3) and Attachment patterns (ECR-GO).
4	Three-week Creation and Sharing Cycle	Three cycles (Home and Group)	Non-verbal access to pre-verbal IWM. Sequential activation and externalization of developmental ego states. Mutual mentalization through group sharing.
5	Post-intervention Assessment	Home-based	Measuring structural changes in IWM through quantitative shifts in Ego States and Attachment patterns.

The specific procedures for each phase are as follows. In the first phase, a two-hour (120 min) orientation session was held online via Zoom for all 31 participants to explain the research procedures and the specific instructions for creating six collage works. Participants were instructed to create collages representing "myself and my surrounding environment" for each of six developmental stages: (1) birth to 18 months, (2) 18 months to six years, (3) seven to 12 years, (4) 13 to 18 years, (5) adulthood before life script modification, and (6) a future after life script modification. The six developmental stages used in this study were adapted from Levin-Landheer's [13] Cycles of Power developmental theory. Levin-Landheer [13] proposed seven developmental stages: Being (birth-6 months), Doing (6-18 months), Thinking (18 months-3 years), Identity (3-6 years), Being Skillful (6-12 years), Regeneration (13-18 years), and Recycling (adulthood). To minimize the burden of collage creation on participants, we consolidated adjacent stages where developmental tasks were conceptually similar and age periods were easily recalled by participants. Specifically, the Being and Doing stages were combined (birth to 18 months); the Thinking and Identity stages were combined (18 months to six years). Being Skillful and Regeneration stages were used as is. During the orientation, participants were encouraged to draw upon photographs from their early years, episodes recounted by family members, and their own reflections when creating collages for periods they could not consciously remember. They were assured that the artistic quality of the work was irrelevant and that they should express their images freely.

In the second phase, a single session of individual psychological counseling (45 minutes per participant) based on TA theory was conducted online via Zoom for each participant. This preliminary assessment of the life script was conducted through a qualitative clinical interview. By integrating the participant’s developmental history and real-time behavioral observations within the TA framework, the therapist identified core script elements such as Injunctions and Early Decisions. The primary objective was to establish a therapeutic alliance, serving as an "external secure base," and to provide a structural baseline for the study before the collage creation process.

In the third phase, following the individual counseling, participants completed psychological assessments at home, including the TEG3 and the ECR-GO.

TEG3 is a standardized instrument based on TA ego state theory that quantitatively measures five ego states: CP, NP, A, FC, and AC [14]. It consists of 50 items, with raw scores ranging from 0 to 20 for each ego state. These raw scores are then converted to T-scores (mean = 50, SD = 10) for clinical interpretation. The validity and reliability of the TEG3 have been well-established in the Japanese population. TEG3 is commercially available (Kaneko Shobo, Tokyo, Japan) and was used under a standard research license. The completion time was approximately 10 minutes.

ECR-GO is an attachment style scale that measures self-image and other-image in attachment contexts with generalized others, rather than specific attachment figures [15]. The scale consists of 30 items, divided into two subscales: Attachment Anxiety (15 items) and Attachment Avoidance (15 items). Items are rated on a 7-point Likert scale ranging from 1 (strongly disagree) to 7 (strongly agree). ECR-GO is freely available for research purposes, and its validity and reliability for measuring adult attachment styles have been previously validated. The completion time was approximately five minutes. The two-dimensional, four-category model of ECR-GO corresponds closely to the "life positions" concept in TA: secure attachment (I'm OK, You're OK), dismissing attachment (I'm OK, You're not OK), preoccupied attachment (I'm not OK, You're OK), and fearful attachment (I'm not OK, You're not OK).

To comprehensively evaluate the structural transformation of the personality, the TEG3 and ECR-GO were analyzed in parallel. In this study, the P and C ego states are hypothesized to correspond to the internal models of others and self, respectively. Therefore, changes in these two scales were compared to examine whether the structural shift in ego states (via TEG3) consistently aligns with the structural transformation of the attachment-based IWM (via ECR-GO). This dual-scale approach was employed to verify the validity of the hypothesized structural equivalence between TA ego states and attachment components.

During the fourth phase, which spanned three weeks, participants engaged in a weekly cycle of creation and sharing. The intervention followed a rigorous weekly cycle involving home-based creation, electronic submission, and a structured online sharing session, as detailed in Table 2. Each week, participants created two collage works at home using the "homework method." After completing each pair of works, they photographed them and sent the images to the researcher. Subsequently, participants presented these works during a three- to four-hour group sharing session via Zoom. This cycle was repeated three times to cover all six developmental stages. In each session, participants presented two of their completed works, explaining what the work represented and the thoughts and feelings embodied in it. Following each presentation, other participants asked questions and offered comments. The therapist served as facilitator, asking questions and providing comments to promote insight as appropriate. During these Zoom sessions, the time allocated specifically for the presentation and discussion of each participant's two works was approximately 20 minutes. After each group sharing session, participants completed a questionnaire asking what they had noticed about themselves and their surrounding environment during the relevant developmental period.

**Table 2:** Detailed procedural flow of the weekly intervention cycle

Stage	Step	Description of Activity
Preparation	1-1	Creation of Collages: The participant creates two assigned collages (e.g., Collage 1 and 2 in Week 1) at home.
	1-2	Electronic Submission: Photographs of the two completed collages are taken and emailed to the therapist.
	1-3	Logistics: The therapist sends a reminder email including the presentation order for the group session.
Group Sharing (Approx. 20 min)	1-4	Participation in Zoom Session: (Structured as follows for each participant)
	1-4-1	Individual Presentation and Dialogue: • Presentation of the first collage for the week (e.g., Collage 1) • Q&A and discussion with other participants regarding that collage • Clinical comments/facilitation from the therapist • Presentation of the second collage for the week (e.g., Collage 2) • Q&A and discussion regarding that collage • Clinical comments/facilitation from the therapist
	1-4-2	Active Observation: When not presenting, the participant observes peers' presentations and voluntarily engages in Q&A.
Reflection	1-5	Post-session Questionnaire: The therapist sends the questionnaire, and the participant completes it regarding insights into their self and environment for the developmental periods represented by the two collages (e.g., birth to six years).

In the final phase, after the third group sharing session, participants again completed TEG3 and ECR-GO at home. Upon completion of all procedures, participants returned the assessment forms, questionnaires, and consent documents to the researcher by mail. The completed collage works were retained by the participants. Written informed consent was obtained from all participants for participation in the research and for publication of their case materials, including collage images.

Case F

Case F was a woman in her 20s employed as an office worker. Her mother was a healthcare professional, and both parents worked. At birth, she lived at her maternal grandparents' home with her great-grandmother and grandparents. Her maternal grandfather was described as a person of great virtue, and her maternal grandmother was caring and very cheerful.

When she entered kindergarten, the family moved to her paternal grandparents' home, and she began living with her paternal grandparents. Her younger brother was born when she was four years old. Her paternal grandmother and mother had a very poor relationship, with constant conflict between them. As a result, the relationship between her parents was also strained. While her mother was busy working, it was her paternal grandmother who primarily cared for her. This grandmother was extremely anxious, constantly telling her, "You must not do this," "You must not do that," and "If this happens, something terrible will happen, something frightening will happen." Around age five, she developed stuttering and became unable to speak words smoothly. Prior to this onset, her speech and language development were reportedly normal and age-appropriate, with no significant concerns regarding fluency. Her grandmother desperately tried to cure the stuttering, and her father frequently criticized and scolded her about her speech. Her relationship with her father gradually deteriorated, and she stopped speaking much at home.

From elementary school, she began attending a "speech classroom" for training to correct her speech. From elementary school years, she was mocked and taunted for her stuttering, and she stopped speaking with anyone except very close friends. Her father also scolded her about her stuttering, and her relationship with him became considerably worse.

Prior to creating the collage works, she completed the pre-intervention assessments. TEG3 scores were: CP, 49; NP, 47; A, 76; FC, 57; and AC, 50. ECR-GO scores were: Attachment Anxiety, 54; and Attachment Avoidance, 51.

Following the intervention protocol, she created six collage works over three weeks.

**Figure 1:**
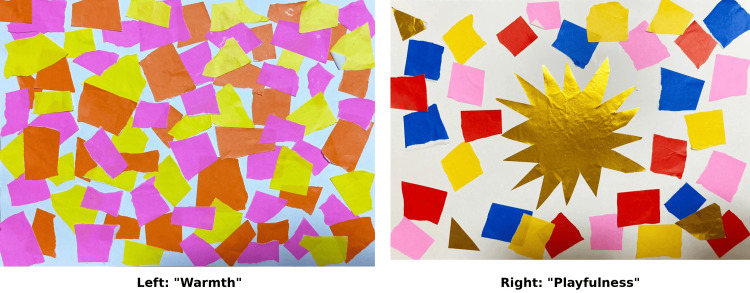
Collages representing Case F's developmental stages (birth to six years) Left: Collage 1 "Warmth" (birth to 18 months). The warm colors (yellow, orange, and pink) represent the Free Child (FC) ego state and a responsive caregiving environment. Right: Collage 2 "Playfulness" (18 months to six years). The gold sun-like shape and vibrant colors symbolize energy, innocence, and the absence of developmental restrictions.

As the first work of Week 1, Collage 1 expressed herself and her surrounding environment from birth to 18 months, and was titled "Warmth" (Figure 1, left).

The work featured yellow, orange, and pink origami paper collaged across the entire surface in a torn-paper style. During the group sharing session, she explained: "Yellow represents mischievousness, orange represents energy, and pink represents love from those around me." Her reflections and insights regarding this developmental stage, as captured in the post-session questionnaire, are presented in Table 3.

**Table 3:** Case F’s responses to the questionnaire after the group sharing session (birth to 18 months)

Items in the Questionnaire	Case F's Responses
Q1. What did you notice about yourself and your surrounding environment up to 18 months?	"I don't have many memories up to 18 months and don't know about my surrounding environment, but I noticed that the memory of being blessed by everyone remained within me as a bodily sensation and image."

As the second work of Week 1, Collage 2 expressed herself and her surrounding environment from 18 months to six years, and was titled "Playfulness" (Figure 1, right). At the center of the work, gold origami paper cut into a sun-like shape was placed. Surrounding it were red, yellow, pink, blue, and gold cut pieces of origami paper. She explained: "Red represents passion, blue represents boyishness, pink represents love and warmth, yellow represents playfulness, and gold represents energy and innocence. I imagined myself during kindergarten. From around age five, signs of stuttering began to appear, but I could still be childlike and innocent." Her reflections and insights regarding this developmental stage are presented in Table 4.

**Table 4:** Case F’s responses to the questionnaire (18 months to six years)

Items in the Questionnaire	Case F's Responses
Q1. What did you notice about yourself and your surrounding environment up to age six?	"Until age six, I was full of energy and was not shut in my shell at all. It became a collage without restrictions, free."

**Figure 2:**
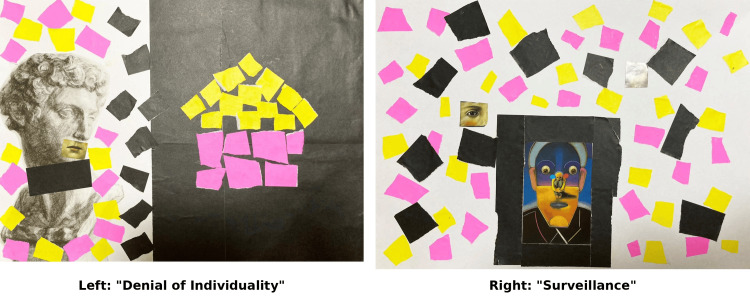
Collages representing Case F's developmental stages (seven to 18 years) Left: Collage 3 "Denial of Individuality" (seven to 12 years). The divided composition and the photo placed on the mouth represent the physical and psychological suppression of stuttering. The black background symbolizes the harsh environment of the speech classroom. Right: Collage 4 "Surveillance" (13 to 18 years). The black border represents a cage of emotional suppression. The scattered "eyes" signify the hypocritical pity and social surveillance felt during adolescence.

As the first work of Week 2, Collage 3 expressed herself and her surrounding environment from ages seven to 12, and was titled "Denial of Individuality" (Figure 2, left).

The work was divided vertically into two halves at the center. On the left side, a plaster statue from the Medici Chapel was pasted on a white background, surrounded by yellow, pink, and black cut pieces of origami paper. On the right side, yellow and pink cut pieces of origami paper were collaged in the shape of a house on black paper. She explained: "The plaster statue figure on the left represents myself, and the rectangular black origami paper on its neck represents suffering and breathlessness. I pasted a realistic photograph on the mouth of this figure to express my stuttering." Regarding the house-shaped origami paper on the right side, she said: "Pink represents intimacy, and yellow represents energy." "The black background expressed the dark environment where I was seen as a pitiable disabled person, and the harsh environment of the speech classroom I began attending at that time, where I was forced to correct my individuality of stuttering." Her reflections and insights regarding this developmental stage are presented in Table 5.

**Table 5:** Case F’s responses to the questionnaire (seven to 12 years)

Items in the Questionnaire	Case F's Responses
Q1. What did you notice about yourself and your surrounding environment up to age 12?	"From entering elementary school until age 12, although my surrounding environment was warm, the speech classroom was a facility that denied my stuttering, so I noticed that walls had formed around me."

As the second work of Week 2, Collage 4 expressed herself and her surrounding environment from ages 13 to 18, and was titled "Surveillance" (Figure 2, right). At the lower center of the work, an illustration of a human figure with a black border was collaged. She explained: "This black frame represents a cage. Inside the cage is a face, and within that face is another person. This person sitting with knees drawn up represents myself. On each shoulder, I collaged red and blue circular cutouts; red represents anger, and blue represents sadness." Outside the black cage were pieces of origami paper scattered about, representing the society and environment surrounding her. She stated: "Pink represents goodwill, yellow represents energy, and black represents failure. Mixed with the origami paper, I collaged human eyes. This represents being watched by various people in society and receiving hypocritical pity. Having stuttering, feeling pity toward myself for failing when I speak, suppressing my emotions, and trying not to talk to people as much as possible - I expressed my former self at that time." Her reflections and insights regarding this developmental stage are presented in Table 6.

**Table 6:** Case F’s responses to the questionnaire (13 to 18 years)

Items in the Questionnaire	Case F's Responses
Q1. What did you notice about yourself and your surrounding environment during middle and high school?	"I noticed that I entered adolescence with walls still around me, increasingly suppressing my feelings of anger and sadness, and trying not to show my emotions."

**Figure 3:**
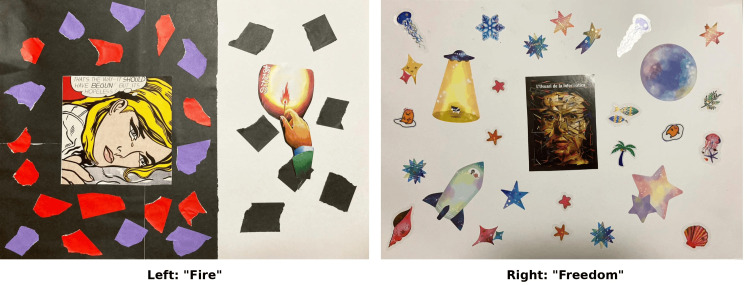
Collages representing Case F's adulthood and future (post-intervention) Left: Collage 5 "Fire" (adulthood). The transition from a black background to a white background, with a small match flame, represents the emergence of self-affirmation amidst previous darkness. Right: Collage 6 "Freedom" (future after life script modification). The scattered colorful imagery and puzzle-like reassembly of the self represent movement, autonomy, and the liberation from the old life script, visually demonstrating the return to a secure-type attachment pattern.

As the first work of Week 3, Collage 5 expressed herself and her surrounding environment as an adult before life script modification, and was titled "Fire" (Figure 3, left).

The work had a black background on approximately the left two-thirds of the drawing paper, and a white background on the right one-third. On the left black paper, an illustration of a crying woman was pasted in the center square, surrounded by purple and red origami paper. According to her, "This illustration of the woman represents myself crying," and "The red origami surrounding me represents anger, and the purple represents a sense of worthlessness." On the right one-third with the white background, she said: "I scattered dark thoughts represented by black origami, and expressed the positive light that began to glow in my heart with the small flame from striking a single match. Through creating this series of collages and the preliminary counseling, I was able to find various thoughts that affirm myself." Her reflections and insights regarding this developmental stage are presented in Table 7.

**Table 7:** Case F’s responses to the questionnaire (adulthood)

Items in the Questionnaire	Case F's Responses
Q1. What did you notice about yourself and your surrounding environment as an adult?	"The walls were thinner than in middle and high school, but things remained dark... However, by lighting the light of self-affirmation, I noticed that I felt the darkness had softened somewhat."

As the second and final work of Week 3, Collage 6 expressed herself and her surrounding environment in the future after life script modification, and was titled "Freedom" (Figure 3, right). At the center of the work, an illustration of a human face was placed, surrounded by space jellyfish, stars, palm trees, rockets, spaceships, and her favorite character scattered about. She explained: "The face illustration in the center represents myself whose life script is beginning to change, myself being rearranged like a puzzle being reassembled, myself with movement. Around me, fun things are flying around very freely."

Following the final group sharing session, Case F reflected on her future and the overall impact of the intervention. Her responses to the final questions in the questionnaire are presented in Table 8.

**Table 8:** Case F’s responses regarding her future and the impact of the intervention

Items in the Questionnaire	Case F's Responses
Q1. What did you notice about yourself and your surrounding environment in the future?	"I noticed that I want to be surrounded by gentle light and animals both inside and outside, and I want to become peaceful, and that I want to become that way."
Q2. What kind of impact, if any, do you think this collage creation has had on your life, mind, or relationships?	"By collaging the situation at that time, it expresses the subconscious rather than language, so I can see things I haven't noticed before. I believe that by doing so, I can change my inner self in my interactions with others and move my relationships in a positive direction."

After the third group sharing session, she completed the post-intervention assessments. TEG3 scores were: CP, 35; NP, 41; A, 76; FC, 59; and AC, 45. ECR-GO scores were: Attachment Anxiety, 48; and Attachment Avoidance, 45. The changes in psychological assessment data are summarized in Table 9.

**Table 9:** Changes in psychological assessment scores before and after intervention TEG3: Tokyo University Egogram Third Edition [14]. Scores are presented as T-scores (mean = 50, SD = 10), where higher scores indicate a stronger tendency for that ego state. CP: Critical Parent; NP: Nurturing Parent; A: Adult; FC: Free Child; AC: Adapted Child. ECR-GO: Experiences in Close Relationships inventory-Generalized Other version [15]. Attachment Anxiety and Attachment Avoidance scores are raw totals based on a 7-point Likert scale (ranging from 1 to 7). TEG3 is commercially available (Kaneko Shobo, Tokyo, Japan) and was used under a standard research license. ECR-GO is freely available for research purposes.

Measure	Pre-intervention	Post-intervention	Change
TEG3			
CP (Critical Parent)	49	35	-14
NP (Nurturing Parent)	47	41	-6
A (Adult)	76	76	0
FC (Free Child)	57	59	2
AC (Adapted Child)	50	45	-5
ECR-GO			
Attachment Anxiety	54	48	-6
Attachment Avoidance	51	45	-6

The post-intervention scores showed a numerical decrease in Attachment Anxiety and Avoidance on the ECR-GO, as well as a more balanced profile in the TEG3 ego states (Table 9). These shifts reflect the quantitative changes in the participant's psychological state following the intervention.

## Discussion

Analysis of individual collage works

The following analysis of individual collage works is not a mere repetition of the clinical findings, but a rigorous theoretical verification process necessary to establish the structural identity of the IWM. This analysis follows a precise deductive logic: (1) verifying whether each collage expresses an "Ego State" as defined by Berne [8]; (2) applying the axiomatic definitions that both collage (Moriya) and IWM (Bowlby [3]) are "mental representations"; and (3) concluding that the identified ego state constitutes a functional component of the IWM. Furthermore, this study maintains high falsifiability; if even one collage work were not identified as an ego state, the hypothesis that "the components of the IWM are ego states" would be rejected.

Collage 1: "Warmth" (Birth to 18 Months)

Case F stated that "the memory of being blessed by everyone remained within me as a bodily sensation and image." According to Bowlby [3], IWM is formed during the pre-verbal period and functions to generate forecasts about the availability and responsiveness of the attachment figure. During this period, Case F is considered to have formed the foundation of a stable IWM within the responsive environment of her maternal grandparents, based on multiple converging lines of evidence: First, developmental history indicates that she was born into and lived with her maternal grandparents, who were described as "caring and very cheerful" (maternal grandmother) and "a person of great virtue" (maternal grandfather), providing a nurturing environment. Second, her phenomenological experience expressed through Collage 1 shows warm colors (yellow, orange, pink representing "love from those around me"), "collaged across the entire surface" without restriction, and she reported an embodied memory of being "blessed by everyone" despite having no conscious memories of this period. Third, the continuity of free expression shown in Collage 2 "Playfulness," where she described herself as "full of energy and was not shut in my shell at all," indicates that the secure attachment foundation established in the earliest period persisted through age six. A search of major academic databases (PubMed, PsycINFO, and CiNii) yielded no other studies on this specific clinical intervention, which integrates TA-based developmental history with longitudinal collage creation, with the exception of the foundational study by Shinoki [9].

In Shinoki's [9] analysis, it was inferred that through creating the collage, Case F re-experienced the "FC" ego state. (Verification: Following Berne’s definition [8] of an ego state as a consistent pattern of feeling and experiencing, the "bodily sensation and image" described by Case F is confirmed as a manifestation of an ego state.) Berne [8] called the earliest layer of the life script the "protocol," stating that it is formed during the nursing period. Erskine [5] noted that these early models (IWM) constitute the unconscious relational patterns that form the life script.

Case F's Collage 1 shows no confusion or conflict in the "C" ego state. This visually indicates that the IWM formed during this period was of the secure type. The choice of colors - yellow (mischievousness), orange (energy), and pink (love from those around) - and the composition in which they are "collaged across the entire surface" are thus considered to express a responsive caregiving environment. Based on the deductive logic of this study, this expression is verified to manifest the FC ego state as the primary component of the early IWM.

Collage 2: "Playfulness" (18 Months to Six Years)

Case F stated that "until age six, I was full of energy and was not shut in my shell at all. It became a collage without restrictions, free." Although signs of stuttering began to appear around age five, she reported that "I could still be childlike and innocent."

During this period, Case F had begun receiving messages from her paternal grandmother, such as "You must not do this" and "You must not do that." However, Collage 2 shows free and unrestricted expression similar to Collage 1. This suggests that although she had begun receiving negative messages, this was a transitional period before the fundamental transformation of the IWM had occurred.

The sun-like gold origami paper placed at the center and the varied colors surrounding it visually express that the FC ego state had not yet been impaired. (Verification: Consistent with the analysis of Collage 1, the unrestricted expression in this work is verified to manifest the persistence of the FC ego state as a component of the IWM during this transitional period.)

Collage 3: "Denial of Individuality" (Seven to 12 Years)

Case F stated that "the speech classroom was a facility that denied my stuttering, so walls had formed around me." This work is divided vertically into two halves at the center, with a plaster statue (representing Case F herself) and black origami paper expressing "suffering and breathlessness" on the white background on the left side, and "the harsh environment" expressed on the black background on the right side.

According to Bowlby [3], IWM is transformed through interactions with attachment figures. During this period, Case F repeatedly experienced negative interactions: criticism from her father, correction in the speech classroom, and curiosity and pity from those around her. Through these experiences, her IWM is considered to have transformed into the insecure type.

In Shinoki's [9] analysis, it was suggested that during this period, Case F received the injunctions "Don't," "Don't trust," "Don't be close," "Don't be important," and "Don't succeed," and life script formation began. It was inferred that through creating the collage, she re-experienced the "AC" ego state of that time. (Verification: Following Berne’s definition [8], this state is identified as the AC ego state responding to external parental restrictions.)

From the perspective of Bowlby's forecasting/simulation function, Case F is considered to have established a prediction pattern during this period that "the other (environment) is critical and negative." Using Berne's protocol concept, Case F formed a transaction pattern of predicting that the other party would be CP and responding with AC. Based on the deductive logic of this study, this prediction-response pattern is verified to function as a structural component of the transformed (insecure) IWM. Collage 3 visually expresses this negative P-C relationship (critical environment and AC ego state).

Collage 4: "Surveillance" (13 to 18 Years)

Case F expressed herself as being "in a cage," experiencing being "watched" and receiving "hypocritical pity," and "suppressing my emotions and trying not to talk to people as much as possible" at that time.

It is shown that the IWM (prediction pattern) formed in Collage 3 was further strengthened during this period. Case F's insight that "I entered adolescence with walls still around me, increasingly suppressing my feelings of anger and sadness, and trying not to show my emotions" indicates that the AC ego state became further fixed.

In Shinoki's [9] analysis, this was described as a process in which the life script was strengthened and justified.

Collage 4 visually expresses the fixed P-C relationship (surveilling environment and suppressed AC). The composition of the black border (cage), the person sitting inside it with knees drawn up (suppressed self), and the eyes scattered around (surveillance) clearly visualizes the predictions based on IWM ("others are critical and intrusive") and the corresponding avoidant behavioral pattern ("trying not to talk"). (Verification: This fixed relationship between a surveilling environment and the suppressed AC ego state is verified to manifest the further reinforcement and fixation of the insecure IWM.)

Collage 5: "Fire" (Adulthood)

Case F stated that "by lighting the light of self-affirmation, I noticed that I felt the darkness had softened somewhat." The composition of the black background (darkness) on the left two-thirds of the work and "the small flame from striking a single match" lit on the white background on the right one-third suggests that the transformation of the IWM had begun.

In Shinoki's [9] analysis, it was inferred that when creating Collage 1, Case F had begun giving herself permission against the injunctions. The reason that script modification began immediately upon creating Collage 5 is considered to be that through creating Collages 1-2, she had re-experienced the FC ego state and was ready to modify her life script.

According to Bowlby [3], IWM can be transformed through new attachment experiences. Through the process of collage creation, Case F is considered to have re-experienced past ego states and begun forming a new P-C relationship (positive internal dialogue). (Verification: Consistent with Bowlby’s theory of IWM plasticity, the emergence of the new positive internal dialogue is verified as the initiation of IWM transformation, structured through the activation of the FC ego state.)

Collage 6: "Freedom" (Future After Life Script Modification)

Case F stated that she "expressed myself whose life script is beginning to change, myself being rearranged like a puzzle being reassembled, myself with movement," and that "around me, fun things are flying around very freely."

In Shinoki's [9] analysis, it was stated that, as the title "Freedom" indicates, the "FC" ego state was expressed. In Collages 1-5, the expression remained "undifferentiated and faltering," with pieces of colored paper torn and specific colors linked to specific emotions and sensations. However, in Collage 6, specific objects such as jellyfish, rockets, and palm trees are freely collaged.

This change in expression visually demonstrates the transformation of IWM. (Verification: As the final step of this deductive study, the free expression of objects is verified to manifest the completion of IWM reorganization into a secure type, characterized by the re-emergence of the FC ego state.) A change can be seen from the prediction patterns of "cage" and "surveillance" expressed in Collages 3-4 ("others are critical," "I should be suppressed") to new prediction patterns of "I may express freely" and "I may be surrounded by fun things."

Changes in psychological assessment data

Examining the changes in TEG3 (Table 2), CP decreased from 49 to 35, NP decreased from 47 to 41, and AC decreased from 50 to 45, while FC increased from 57 to 59. A remained unchanged at 76.

In Shinoki's [9] analysis, it was considered that because Case F gave herself permission in Collage 1 "Warmth" that she did not need to follow the injunctions, the negative "CP" internalized from having her stuttering denied at the speech classroom - expressed in the black background on the right half of Collage 3 "Denial of Individuality" - and the negative "NP" associated with hypocritical pity toward her stuttering both decreased. Additionally, "AC" decreased due to the positive light kindled in her heart, expressed in the right one-third of Collage 5 "Fire," and "FC" increased in Collage 6 "Freedom."

Examining the changes in ECR-GO, Attachment Anxiety decreased from 54 to 48, and Attachment Avoidance decreased from 51 to 45. In Shinoki's [9] analysis, it was considered that Attachment Anxiety decreased because self-affirmation increased through the redecision to modify the script, and Attachment Avoidance decreased because the self and the surrounding environment also changed to something free, enjoyable, and positive.

These changes in psychological assessment data are consistent with the transformation of IWM visualized in the collage works.

General discussion

To my knowledge, this is the first case report to visualize the components of IWM through collage works and to demonstrate changes in both ego state measures and attachment measures before and after intervention. The absence of prior research may be attributed to the fact that no one had assumed it possible to visualize IWM, which is a mental representation. Such an approach requires integration of two distinct theoretical frameworks - attachment theory and TA - as well as the use of a non-verbal technique with appropriate instructions capable of accessing and externalizing mental representations from pre-verbal developmental periods.

Clarification of IWM Components

Analysis of this case suggests that ego states may be included among the components of IWM. Bowlby [3] described IWM as an internal structure that generates forecasts about the availability and responsiveness of attachment figures, but did not clarify its specific components. Meanwhile, Berne [8] stated that the first script programming takes place during the nursing period, in the form of short protocols which can later be worked into complicated dramas. Erskine [5] described the script protocol as preverbal, subsymbolic, physiological, and affective survival reactions - the earliest formations of unconscious relational patterns. He noted that these protocol-based IWM constitute the core of life scripts, integrating attachment theory with script theory. The "short protocols" described by Berne [8] can thus be understood as the infant's patterned responses to caregiver ego states: responding with FC when the caregiver is perceived as NP, or with AC when perceived as CP.

In this case, Case F's collage works visually expressed the relationship between ego states - FC and AC - at each developmental stage and the surrounding environment (P). Collages 1-2 expressed the FC ego state, Collages 3-5 (left side) expressed the AC ego state, and Collages 5 (right side) through 6 again expressed the FC ego state. The arrangement and changes in these ego states are considered to reflect the formation and transformation of prediction patterns based on IWM.

Forecasting/Simulation Function and Ego States

According to Bowlby [3], IWM generates forecasts about the availability and responsiveness of attachment figures. This forecasting function corresponds to Berne's protocol concept.

Children observe the non-verbal messages of caregivers and predict what kind of response they can expect. Based on these predictions, they switch their ego state to FC or AC in order to maintain complementary transactions. For example, in Case F's situation, during the period of Collages 3-4, she established the pattern of predicting that "the other is CP (critical)" and "responding with AC (adapted)."

The structure that carries this prediction-response pattern is IWM, and ego states are considered to function as its components.

Visualization and Transformation of IWM

This case suggests that TA Developmental Collage Therapy may be able to visualize IWM and facilitate its transformation. Collage works visually express the relationship between self and environment at each developmental stage without the mediation of language. As Bowlby [3] pointed out, much of IWM is formed during the pre-verbal period, so it may not be fully accessible through verbal approaches alone. Collage, as a non-verbal technique, is considered to complement this limitation.

Two main factors can be considered for the transformation of IWM. First, as an external factor, there is a change in attachment figures. Second, as an internal factor, there is autonomous change in the relationships among the "Parent," "Adult," and "Child" ego states.

In this case, external factors are clearly expressed in Collages 3-4. Case F's attachment figures changed from her responsive maternal grandparents to her critical paternal grandmother and father. This change in external attachment figures brought about the transformation of IWM to the insecure type.

On the other hand, transformation through collage creation is considered to be primarily due to internal factors. Through the present "A" ego state understanding each ego state that constitutes one's personality and its developmental history, the P-C relationship that had been generating conflict was transformed - the relationship between the critical environment and the adapted self expressed in Collages 3-4.

Through this internal transformation, P-A-C began to function harmoniously, and integration of the personality as a whole was facilitated. This is considered a sign of change toward secure attachment. Collage 6 "Freedom" can be understood as a prediction that this integrated personality will function cooperatively in future interpersonal relationships.

Bowlby [3] suggested that IWM is updated as cognitive development progresses. If P-A-C are considered to be components of IWM, then IWM is thought to have a self-updating function in which the "A" ego state regulates the relationship between the "P" and "C" ego states.

However, when the P-C relationship becomes rigid for some reason, this self-updating function is thought to decline. Critical caregiving environments can be cited as a cause. In this case, the rigidification of the CP-AC relationship expressed in Collages 3-4 can be interpreted as a visualization of precisely this state.

TA Developmental Collage Therapy can be understood as a method that regulates relationships among the components of IWM and promotes recovery of the self-updating function. In this case, the transformation of IWM occurred not through a new attachment figure from outside but through an internal process via collage creation. This suggests that the self-updating function of IWM was recovered.

An important feature of TA Developmental Collage Therapy is that by tracing back through developmental stages, it becomes possible to visualize when IWM was transformed. In this case, it was visually demonstrated that stable IWM in Collages 1-2 transformed to the insecure type in Collage 3. It was stable from birth to age six, and transformed between ages seven and 12. This makes it possible to identify the depth of the wound, that is, at which developmental stage IWM was transformed.

Relationship to Contemporary Attachment Theory

Bowlby [3] stated regarding the function of IWM that "a person perceives events, forecasts the future, and constructs plans." He also pointed out that "human beings do not live only in the present. As a child's cognitive capacities develop, he becomes able to foresee the occurrence of various situations." This forecasting function is derived from models formed based on past experience.

Bowlby's theory has subsequently come to be understood in relation to concepts such as "attunement" by Stern [16] and "mentalization" by Fonagy and colleagues [17].

Regarding attunement, Stern [16] pointed out the importance of affect attunement, in which caregivers attune to infants' internal states. Bowlby [3] also stated that "the model of the attachment figure and the model of the self tend to develop complementarily and mutually confirming." Conventionally, attunement has been understood as occurring between caregiver and infant, and in treatment settings for attachment difficulties, as a process required between therapist and patient [16]. However, in this therapy, the present "A" ego state attunes to the ego states of each developmental stage through collage creation. This is not attunement by an external other but autonomous attunement within the self. Furthermore, because this attunement process is concretely externalized as collage works, it becomes possible to consciously and objectively access the complementary models formed in the past through visual means.

Regarding mentalization, Fonagy and colleagues [17] systematized the concept of mentalization - the capacity to understand one's own and others' mental states - integrating it with attachment theory. Bowlby [3] identified as therapeutic goals "detecting the existence of influential models of which the patient is partially or completely unaware" and "having the patient examine those models and consider whether they are still valid." In this therapy, by responding to the question "What did you notice about yourself and your surrounding environment?" after creating each collage, awareness of the individual ego states that constitute one's personality and the history of personality formation is promoted. This is precisely the process of mentalization. And through the group sharing component of this therapy, participants can see the contents and formation history of others' personalities through their collage works, and by hearing the accompanying narratives directly from the individuals themselves, the effects of mentalization are considered to be further enhanced.

Regarding the visualization of IWM, Bowlby [3] proposed the concept of "working models of attachment figures and of the self," but did not address methods for visualizing them. This therapy makes it possible to externally represent IWM, which is difficult to verbalize, through the non-verbal technique of collage. This is a unique contribution of this therapy in that it concretely externalizes what has conventionally been a representational concept.

Bowlby [3] stated regarding the importance of the temporal dimension in development: "Until about three years of age, actual presence/absence is the dominant variable. After three years, forecasts about availability or unavailability become increasingly important." TA Developmental Collage Therapy has a structure that sequentially accesses IWM at each developmental stage along a temporal axis from birth to the present and into the future. This structure along the temporal axis makes it possible to visualize and re-examine the process of "forecast" formation that Bowlby described.

As described above, by systematically externalizing mental representations through the creation and sharing of developmental collages, including the pre-verbal period, TA Developmental Collage Therapy provides unique contributions of IWM visualization and autonomous attunement. This intervention specifically targets the transformation of maladaptive IWMs - manifested as avoidant behaviors or emotional suppression - into secure attachment patterns characterized by free expression. In doing so, it encompasses the attachment theory proposed by Bowlby and the related concepts of Stern, Fonagy, and others.

Development and methodological approach

Addressing a Fundamental Gap in Attachment Theory

Attachment theory identifies IWM as central to understanding attachment-related difficulties, yet what IWM structurally consists of has never been defined. This definitional gap has impeded both theoretical development and clinical application for over 50 years. This research tests whether TA concepts - specifically, ego states - can provide the missing structural framework. Erskine's [5] proposal that life scripts and IWM are conceptually equivalent provides the theoretical bridge enabling this cross-theoretical application.

The Staged Development of Integration

The doctoral work [9] combined two hypotheses: (A) ego states are included among the components of life scripts (inferred from Berne's [8] script matrix diagram), and (C) collage therapy externalizes non-verbal mental representations (Moritani [12]). The doctoral research (n=31) demonstrated that developmental collages enable life script visualization, analysis, and modification.

The present research adds hypothesis B: life scripts and IWM are conceptually equivalent (Erskine [5]). By combining all three hypotheses (A + B + C), this study reanalyzes collage works from the doctoral research within the framework of attachment theory, testing whether the same visualization method enables structural identification of IWM components as ego states.

Why the Integration Was Theoretically Possible

The integration of these three hypotheses creates a deductive chain: If ego states are components of scripts (A), and scripts are equivalent to IWM (B), and collage externalizes mental representations (C), then developmental stage collages should enable visualization and structural identification of IWM components as ego states. This deductive chain is falsifiable: if even one developmental collage failed to correspond to an ego state, the entire hypothesis chain would be rejected. The systematic correspondence observed across all six collages in Case F retrospectively validates the chain.

The Methodological Pathway

Through 23 years of clinical practice using regression hypnosis with the ego state model (approximately 3,000 cases), limitations became apparent: high invasiveness and unsuitability for group therapy. This led to exploring collage therapy as a less invasive alternative. The doctoral research (2017-2021) validated that hypotheses A + C enable life script visualization. The present case report adds hypothesis B, demonstrating that the same method enables IWM component visualization - a proof of concept that what has eluded the field for over 50 years is achievable through this theoretical integration.

Why No Prior Research

The absence of prior research reflects that the doctoral work itself (combining A + C) had no precedent. Adding hypothesis B transforms the method from "life script visualization" to "IWM component visualization" - a conceptual leap requiring theoretical deduction (Erskine's integration), extensive clinical foundation (23 years), methodological innovation (developmental collage framework), and systematic validation (doctoral research, 31 participants). Most clinical research follows purely inductive pathways (technique first, theory later), whereas this study followed deductive-to-inductive progression across two stages: doctoral work establishing A + C, then this study adding B for reanalysis.

Theoretical implications for attachment theory

The findings of this case study have implications for attachment theory and its clinical applications. First, the observation that collage works depicting the relationship between the "C" ego state and the surrounding environment ("P") correspond to Bowlby's definition of IWM suggests a potential structural equivalence between TA ego states and IWM components. This supports Erskine's [5] theoretical integration and extends it by providing visual evidence.

Second, the transformation observed through collage creation occurred without the involvement of external attachment figures, challenging the traditional assumption that IWM modification requires new relational experiences with others. The "FC" ego state expressed in Collages 1 and 2 suggests that warm transactions with the "NP" of the maternal grandparents existed in the early environment. This early relational experience served as an internal secure base, facilitating the reorganization of IWM. This finding is consistent with Brown and Elliott's [18] concept of "ideal parent figure protocol."

Third, the non-verbal nature of collage therapy may provide unique access to pre-verbal IWM formations. Schore [4] emphasized that early attachment experiences are encoded in implicit, procedural memory systems that are difficult to access through language. The collage technique, by bypassing verbal processing, may directly access these implicit representations.

Limitations and future directions

This study has several limitations. First, as a single case study, caution is required in generalizing the results. Second, the claims in this study regarding the components of IWM are based on theoretical inference and have not been directly verified. Third, because there is no long-term follow-up data, the persistence of the transformation remains unknown. Future directions include verification of the reproducibility of results through analysis of multiple cases, more detailed theoretical examination of the components of IWM, and long-term follow-up studies.

Furthermore, the ego state model of TA may provide a promising hypothesis for explaining mind-body correlations. Berne defined ego states as "coherent systems of thought and feeling manifested by corresponding patterns of behavior" [8], which inherently includes physiological responses. Future research should explore how the fixation of specific ego states (such as over-adapted AC) relates to somatic symptoms and psychosomatic conditions.

To establish evidence-based support for this therapeutic approach, future studies should employ standardized psychological assessment instruments such as TEG3 [14] and ECR-GO [15] with larger sample sizes. Such quantitative research would enable statistical validation of the relationship between ego state changes and attachment pattern modifications, thereby contributing to the integration of TA with mainstream clinical psychology.

## Conclusions

This case report demonstrated that TA Developmental Collage Therapy can visualize IWMs and facilitate their transformation. Analysis of collage works created across six developmental stages revealed that IWM may comprise ego states as structural components. The collages visually expressed the relationship between the "C" ego state (FC or AC) and the surrounding environment ("P"), corresponding to Bowlby's definition of IWM as consisting of "a model of the attachment figure" and "a model of the self."

The transformation observed in this case occurred through an internal process - the present "A" ego state attuning to past ego states through collage creation - rather than through external attachment figures. Changes in psychological assessment data (TEG3 and ECR-GO) were consistent with the visual transformation depicted in the collage works. These findings suggest that combining non-verbal collage techniques with verbal approaches may provide an effective therapeutic framework for accessing and modifying IWM formed during the pre-verbal period.
